# Risk Factors for Mortality in *Candida auris* Bloodstream Infection: A Multicenter Study in South Korea, 2018–2025

**DOI:** 10.3390/jof12070519

**Published:** 2026-07-15

**Authors:** Min Han, Jin Young Ahn, Jae Eun Seong, Se Ju Lee, Jinnam Kim, Jae Hoon Kim, Joon Sup Yeom, Jun Yong Choi, Su Jin Jeong, Jung Ho Kim, Jung Ah Lee, Yong Seop Lee, Hyuk Min Lee, Nam Su Ku

**Affiliations:** 1 Division of Infectious Diseases, Department of Internal Medicine, Yonsei University College of Medicine, Seoul 03722, Republic of Korea; hanmin2025@yuhs.ac (M.H.); comebacktosea@yuhs.ac (J.Y.A.); jaehoon0215@yuhs.ac (J.H.K.); joonsup.yeom@yuhs.ac (J.S.Y.); seran@yuhs.ac (J.Y.C.); jsj@yuhs.ac (S.J.J.); qetu1111@yuhs.ac (J.H.K.); peacefulee@yuhs.ac (J.A.L.); yslee@yuhs.ac (Y.S.L.); 2 Division of Infectious Diseases, Seoul Medical Center, Seoul 02053, Republic of Korea; sjaeeun1127@yuhs.ac; 3 Division of Infectious Diseases, Inha University College of Medicine, Incheon 22332, Republic of Korea; playit@inha.ac.kr; 4 Division of Infectious Diseases, Hanyang University College of Medicine, Seoul 04763, Republic of Korea; jam764@hanyang.ac.kr; 5 Division of Laboratory Medicine, Yonsei University College of Medicine, Seoul 03722, Republic of Korea

**Keywords:** *Candida auris*, bloodstream infection, mortality, risk factors

## Abstract

*Candida auris* (*C. auris*) is a multidrug-resistant pathogen that spreads clonally in healthcare settings and was designated an urgent threat by the Centers for Disease Control and Prevention in 2019. We conducted a multicenter study to identify mortality risk factors in *C. auris* bloodstream infection (BSI) in South Korea. In this retrospective cohort study across three tertiary centers, 50 adults with first-episode *C. auris BSI* were analyzed. Primary and secondary outcomes were 30- and 90-day mortality. 30-day mortality was 24%, and 90-day mortality was 46%. Older age and higher Sequential Organ Failure Assessment score were independently associated with 30-day mortality. Microbiologic clearance within 30 days was protective. For 90-day mortality, older age was the only independent risk factor, and microbiologic clearance within 90 days was also protective. In conclusion, *C. auris* BSI causes substantial 30-day mortality, underscoring the need for risk-stratified, clearance-focused management.

## 1. Introduction

*Candida auris* (*C. auris*) was first identified in Japan in 2009, and retrospective investigations have traced earlier isolates back to 1996 in South Korea [1]. Because of its ability to adhere to skin, medical devices and environmental surfaces and its resistance to multiple antifungal agents, *C. auris* causes nosocomial transmission, leading to multiple outbreaks, and rapidly emerged as an “urgent threat” in the 2019 Antibiotic Resistance Threats Report of the Centers for Disease Control and Prevention (CDC) [2].

Unlike other Candida species, in which endogenous translocation is the predominant route of infection, *C. auris* spreads through clonal transmission facilitated by persistent skin colonization, biofilm formation on medical devices, and prolonged survival on healthcare environmental surfaces [3]. Consequently, transmission frequently occurs within healthcare facilities, making rapid case recognition and infection prevention essential [3,4,5]. Current CDC guidance recommends active surveillance of high-risk contacts, implementation of contact precautions or enhanced barrier precautions according to healthcare settings, dedicated patient-care equipment, and thorough daily and terminal environmental disinfection to limit transmission [6]. Nevertheless, despite these extensive infection prevention efforts, colonized patients—particularly those requiring invasive devices, intensive medical care and immunocompromised—remain at risk for progression to invasive infection, which is associated with substantial morbidity and mortality [3,7,8].

Although echinocandins remain the recommended first-line therapy for invasive *C. auris* infection, clinical management remains challenging because interpretation of antifungal susceptibility testing is complicated and clinical breakpoints have undergone recent updates [9]. In addition, considerable variability in antifungal resistance, virulence-associated traits, and phenotypic characteristics—including biofilm formation and environmental persistence—may contribute to the heterogeneous clinical outcomes reported across studies [5,10]. Recent experimental data have shown that *C. auris* exhibits enhanced virulence at physiological and febrile temperatures, characterized by increased thermotolerance, antifungal MICs, melanin production, hemolytic activity, and adhesion, supporting its successful adaptation and increased virulence to the human host [11]. These biological and therapeutic complexities underscore the need to identify robust clinical predictors of mortality that may improve risk stratification and guide management beyond current guideline-based antifungal therapy.

Once *C. auris* colonizes human surfaces or the healthcare environment and progresses to invasive infection, particularly bloodstream infection (BSI), outcomes are poor. Reported mortality for *C. auris*–associated BSI ranges from 33% to 72% across studies [12,13]. Although some studies have suggested that crude mortality of *C. auris* BSI is comparable to that of BSI caused by other *Candida* species (30-day, 25%; 90-day, 37%), a study from New York reported substantially higher 30- and 90-day mortality rates of 39% and 58%, respectively [12,13,14,15,16,17]. These wide discrepancies likely reflect the small number of studies designed primarily to evaluate mortality, differences in baseline severity between comparison groups within each study, and the lack of consistent adjustment for both acute severity (Sequential Organ Failure Assessment [SOFA] score) and chronic comorbidity burden (Charlson Comorbidity Index [CCI]). In addition, heterogeneity in clinical variables collected and differences in the predominant circulating clades included in each cohort make it difficult to define the “true” mortality of *C. auris* BSI. Furthermore, studies specifically addressing mortality risk factors in patients with *C. auris* BSI remain scarce, and the determinants of poor outcomes are still not fully understood [12,15,17].

In Korea, the isolation of *C. auris* from non-ear specimens, particularly blood and urine, is increasing, and highly virulent clade I strains have recently been detected among bloodstream isolates [1,18,19,20]. However, no Korean data specifically evaluating mortality and its determinants in patients with *C. auris* BSI are available.

Given the paucity of studies that have consistently evaluated mortality risk factors in *C. auris* bloodstream infections and the absence of such data from East Asian countries, including Korea, we conducted a multicenter study to describe the clinical characteristics of *C. auris* BSI and to identify risk factors for mortality.

## 2. Materials and Methods

### 2.1. Study Population

This retrospective multicenter cohort study included patients admitted to three tertiary care centers (Sinchon Severance Hospital, Inha University Hospital, and Hanyang University Hospital) between 1 January 2018 and 1 June 2025. We included patients who had a first episode of *C. auris* infection identified from blood culture. Patients younger than 18 years and those with concomitant bacteremia were excluded.

### 2.2. Variables and Definitions

Clinical data were retrospectively extracted from electronic health records, including demographics, medical comorbidities, presence of invasive medical devices, prior antibiotic and antifungal exposures, documented sources of infection, severity-of-illness indices (Pitt bacteremia score, SOFA score, and CCI, such as chronic comorbidity burden); BSI status, and clinical outcomes. BSI status was categorized as persistent (consecutive blood cultures remaining positive ≥48 h after initiation of appropriate therapy), breakthrough (*C. auris* BSI occurring after documented microbiological clearance, defined as two consecutive negative cultures, while systemic antifungal therapy was still ongoing), relapse (*C. auris* BSI after documented microbiological clearance following discontinuation of antifungal therapy), and microbiological resolution (sustained documented clearance without breakthrough or recurrence). Follow-up blood cultures were obtained at the discretion of the treating physician, generally every 24–48 h until bloodstream clearance was documented. Appropriate antifungal therapy was defined according to antifungal susceptibility testing results. Catheter-related bloodstream infection was diagnosed based on the treating physician’s assessment considering microbiological findings, the absence of an alternative source, catheter management, and the clinical course, and source control was considered achieved when the presumed focus was appropriately managed. The primary outcome was 30-day all-cause mortality, and the secondary outcome was 90-day all-cause mortality. The objective of this study was to identify independent predictors of 30- and 90-day mortality in patients with *C. auris* BSI.

### 2.3. Microbiological Tests

All clinical isolates were identified as *C. auris* using Matrix-Assisted Laser Desorption/Ionization–Time of Flight (MALDI-TOF) (VITEK MS, bioMérieux, Marcy l’Étoile, France) mass spectrometry, and antifungal susceptibility testing (AFST) was performed using the Sensititre YO10 yeast susceptibility microbroth dilution plate (Thermo Fisher Scientific, Cleveland, OH, USA) according to the manufacturer’s instructions, based on the CLSI M27 broth microdilution reference methodology. The determined MICs were interpreted using the CDC tentative breakpoints for *C. auris* [21].

For genomic characterization, genomic DNA was extracted from each *C. auris* isolate using the DNeasy Blood & Tissue Kit (Qiagen, Hilden, Germany). Whole-genome sequencing (WGS) was performed on the Illumina NovaSeq 6000 platform (Illumina, San Diego, CA, USA) with paired-end 150 bp reads, targeting a minimum coverage depth of X50. Raw sequencing reads underwent quality filtering and adapter trimming using Trimmomatic (v0.39.06). De novo genome assembly was carried out using SPAdes (v3.15.5), and assembly quality metrics (N50, total assembly length, number of contigs) were assessed with QUAST (v5.2.0). Assembled genome sequences were submitted to Pathogenwatch, a web-based genomic epidemiology platform, for clade designation [22]. Clade assignment was based on core-genome single-nucleotide polymorphism (SNP) analysis against the established *C. auris* reference genomes representing Clades I–V, as implemented within the Pathogenwatch *C. auris* typing scheme.

### 2.4. Statistical Analysis

Categorical variables were compared using Fisher’s exact test or the Pearson χ^2^ test as appropriate, and continuous variables were compared using Student’s *t*-test or the Mann–Whitney U test, as appropriate, according to the distribution of the data. Multivariate analyses were performed using logistic regression in a backward, stepwise, conditional manner. Variables included in the multivariable model were selected based on clinical relevance, prior literature, and variables with *p* < 0.10 in univariable analysis to account for potential confounding. Odds ratios (ORs) and 95% confidence intervals (CIs) were calculated. We compared risk factors between 30-day non-survivors and survivors, and between 90-day non-survivors and survivors. Variables showing statistically significant associations were visualized using Kaplan–Meier survival curves. All tests of significance were two-tailed, and statistical significance was set at *p* < 0.05. Statistical analyses were performed using SPSS statistics software (version 18.0; SPSS Inc., Chicago, IL, USA).

### 2.5. Ethical Approval

This study was approved by the University of Yonsei Institutional Review Board (IRB) as the primary IRB (#2025-3853-001) and by the IRBs of the participating institutions. The requirement for informed consent was waived due to the retrospective nature of the study.

## 3. Results

**Figure 1:**
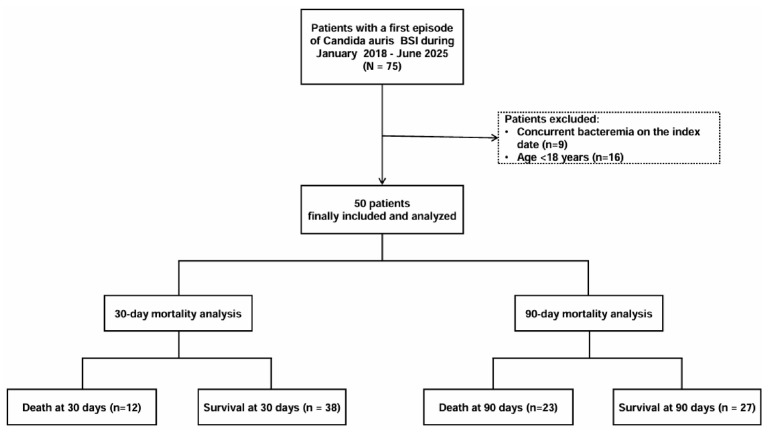
Flowchart of patient selection for 30-day and 90-day mortality analyses.

Among the 75 patients screened across the hospitals, 50 met the inclusion criteria after excluding those with concurrent bacteremia (*n* = 9) and those aged < 18 years (*n* = 16). The final analytical cohort of 50 patients was divided into 30-day and 90-day survivors and non-survivors (Figure 1).

The analytical cohort comprised 38 patients from Yonsei Severance Hospital, 11 from Inha University Hospital, and one from Hanyang University Hospital. Of the 50 patients with *C. auris* BSI, 66% were male, and 70% were ≥65 years of age. Antifungal susceptibility testing was performed on all isolates (n = 50), and all had susceptibility to echinocandins.

The primary outcome, 30-day all-cause mortality, was 24% (12/50) and the secondary outcome, 90-day all-cause mortality, was 46% (23/50) (Table 1). Table 1 presents detailed cohort characteristics stratified by survival status on day 30 and day 90. 30-day decedents had higher SOFA scores (mean ± SD, 11.42 ± 5.50 vs. 6.29 ± 4.36, *p* = 0.002), more frequent *C. auris* colonization (75.0% vs. 36.8%, *p* = 0.021), and were less likely to achieve microbiological resolution (25.0% vs. 71.1%, *p* = 0.007) by day 30. 90-day decedents were older (mean± SD, 75.52 ± 8.91 vs. 65.00 ± 17.43, *p* = 0.017) and had higher Pitt bacteremia scores (mean ± SD, 4.57 ± 2.48 vs. 3.11 ± 2.21, *p* = 0.033) and SOFA scores (mean ± SD, 9.91 ± 5.46 vs. 5.40 ± 8.81, *p* = 0.002); and were less likely to achieve microbiological resolution (47.8%vs 88.9%, *p* = 0.002).

In multivariable analyses, older age (adjusted odds ratio [aOR], 1.09; 95% CI, 1.01–1.19; *p* = 0.045) and higher SOFA score (aOR, 1.12; 95% CI, 1.01–1.42; *p* = 0.040) were independently associated with 30-day mortality, whereas microbiological clearance within 30 days was protective (aOR, 0.11; 95% CI, 0.02–0.69; *p* = 0.019) (Table 2). At 90 days, age was an independent factor associated with increased odds of 90-day mortality (aOR, 1.08; 95% CI, 1.01–1.17; *p* = 0.027), and, similar to the 30-day analysis, microbiological clearance within 90 days was a protective factor associated with decreased odds of death (aOR, 0.169;95% CI, 0.028– 0.996; *p* = 0.049) (Table 2).

As a sensitivity analysis, simplified multivariable logistic regression models including only age, SOFA score, and microbiological clearance were constructed. In these analyses, older age (adjusted odds ratio [aOR], 1.09; 95% CI, 1.01–1.19; *p* = 0.039) and microbiological clearance within 30 days (aOR, 0.15; 95% CI, 0.03–0.76; *p* = 0.028) remained independently associated with 30-day mortality, whereas SOFA score was not. Similarly, at 90 days, older age (aOR, 1.09; 95% CI, 1.02–1.18; *p* = 0.021) and microbiological clearance within 90 days (aOR, 0.16; 95% CI, 0.02–0.89; *p* = 0.046) remained independently associated with mortality, while SOFA score was not (Table 3).

**Figure 2:**
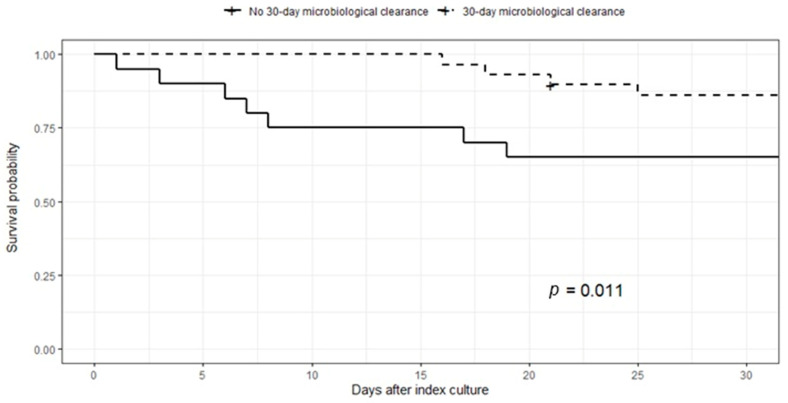
Kaplan-Meier survival curve according to 30-day microbiological resolution of BSI; crosses indicate censored data.

Kaplan–Meier curves of 30-day overall survival from the index culture, stratified by BSI resolution within 30 days, diverged within the first month and remained widely separated throughout follow-up. Patients achieving clearance by day 30 had substantially higher survival, stabilizing near the high-80% range, whereas those without clearance experienced early death, followed by a plateau around the mid-50% range (Figure 2).

## 4. Discussion

In our study, 30-day mortality was 24% (12/50); 90-day mortality was 46% (23/50). In multivariate models, older age and microbiological clearance were independently associated with both 30-day and 90-day mortality, whereas a higher SOFA score was independently associated only with 30-day mortality.

*C. auris* BSI is associated with mortality rates ranging from 33% to 72% [12,14,23]. While in the study by Chen et al., the 30- and 90-day mortality was reported as approximately 20–45%, which was comparable to that observed for drug-resistant bacterial BSIs, a study conducted mainly in New York by Adams et al. reported 30- and 90-day mortality rates of 39% and 58%, respectively [13,15]. Another study that compared BSI caused by *C. auris* with that caused by other *Candida* species showed 30- and 90-day mortality rates of 25% and 37%, respectively, and concluded that *C. auris* BSI was not associated with higher mortality than non–*C. auris* BSI [24]. However, these findings were limited to echinocandin-susceptible isolates, and the non–*C. auris* group had significantly higher ICU admission rates and SOFA scores at baseline, which may have led to an underestimation of the true mortality associated with *C. auris*.

In our study, the 30-day and 90-day mortality rates were 24% and 46%, respectively. All isolates belonged to clade I, and antifungal susceptibility testing showed 96% resistance to fluconazole, approximately 80% resistance to amphotericin B, and complete susceptibility to echinocandins, which was broadly consistent with the previously reported antifungal susceptibility pattern of clade I, except for amphotericin B susceptibility [15,19]. In our study, all but one isolate demonstrated echinocandin MICs of ≤0.25 mg/L; the remaining isolate had an MIC of 0.5 mg/L. Recently, EUCAST established species-specific clinical breakpoints for *C. auris*. Reassuringly, all isolates in our study remained susceptible to echinocandins according to these updated interpretive criteria. In contrast, the relatively high amphotericin B MICs observed in our cohort should be interpreted in the context of recent EUCAST guidance, which notes that amphotericin B susceptibility testing may be influenced by the testing methodology employed [9]. Similar to the findings of Simon et al., echinocandin-susceptible *C. auris* BSIs may exhibit mortality rates comparable to those of BSIs caused by other *Candida* species, but are characterized by a higher microbiological recurrence rate [24]. Thus, even if short-term mortality appears similar, inadequate infection control may allow *C. auris* to become endemic, resulting in a cumulative increase in infection episodes and deaths over time. Given that *C. auris* is on the verge of being designated as a notifiable infectious disease in Korea, the fact that its mortality rate does not exceed that of other *Candida* species should not lead to the underestimation of *C. auris* as an urgent and clinically important pathogen [25,26]. Notably, *C. auris* colonization was more frequently observed among 30-day non-survivors in our cohort. Although this finding does not establish a causal relationship, it underscores the potential clinical significance of colonization and highlights the importance of active surveillance and strict infection control measures to prevent transmission and subsequent invasive infection [1,3,4,18].

Historically, Korea has been regarded as a region wherein clade II strains are predominant. However, in 2022, a clonal outbreak of *C. auris* clade I BSI occurred in association with a COVID-19 patient imported from Vietnam, and the clinical infections caused by clade I became increasingly prominent [1,18,27]. Echinocandin-non-susceptible clades may be introduced into Korea at any time, and such strains would be expected to confer even higher mortality. Therefore, *C. auris* should remain a target for aggressive infection control measures, and continuous accumulation of clinical and microbiological data is required.

This study evaluates mortality risk factors for *C. auris* bloodstream infection by incorporating clade-level genomic analysis and provides data from the East Asian region. A recent study on *C. auris* did exist; however, it defined mortality in a way that included transfer to hospice care, making it difficult to interpret the results as a direct evaluation of mortality, and it did not include clade-level genomic typing [17,28]. Our dataset, which integrates antifungal susceptibility results with genomic information, is expected to provide important guidance for managing infections caused by clade I *C. auris* in the future.

In our study, microbiological eradication was an important determinant of early mortality, consistent with previous reports. Arlan et al. identified fungal eradication and SOFA scores as key risk factors for mortality in patients with *C. auris* BSI [29]. In a large cohort study evaluating the factors associated with BSI resolution, the presence of a hemodialysis catheter was associated with reduced odds of BSI resolution. Also, Fan et al. showed that invasive devices are major risk factors for *C. auris* infection and require careful management as part of infection control strategies [5]. In light of our finding that microbiological resolution is associated with a lower risk of mortality, future studies should focus on identifying the factors that facilitate or hinder microbiological clearance [17].

This study had several limitations. First, because of its retrospective nature, causality cannot be definitively established. Second, assessment of microbiological clearance is subject to immortal time bias, as patients must survive until the assessment time point to be classified as having achieved clearance. Although we evaluated clearance at prespecified time points, residual bias may remain. Future studies using landmark analysis or time-dependent approaches are warranted to better address this issue. Although this limitation precludes a causal interpretation of the relationship between microbiological clearance and mortality, our findings nevertheless suggest that clearance may be an important prognostic factor associated with improved survival. Importantly, unlike fixed patient characteristics such as age or baseline comorbidities, microbiological clearance represents a potentially modifiable clinical target through appropriate therapeutic interventions. Therefore, efforts to optimize and accelerate bloodstream infection (BSI) clearance may have meaningful clinical implications. Further studies are warranted to identify strategies that promote microbiological clearance and to evaluate whether interventions aimed at achieving earlier clearance can translate into improved patient outcomes in real-world clinical practice. Finally, the relatively small sample size limited the generalizability of our results. We plan to address this limitation through future multicenter studies in Korea that are based on the present dataset.

## 5. Conclusions

In conclusion, *C. auris* BSI was associated with substantial mortality. Older age, higher SOFA scores, and failure to achieve early microbiological clearance independently predicted mortality, underscoring the need for risk-stratified, clearance-focused management.

## Figures and Tables

**Table 1:** Baseline characteristics of patients with *Candida auris* bloodstream infection by 30-day and 90-day survival status, South Korea, 2018–2025.

	30-Day Survivors (*n* = 38)	30-Day Decedents (*n* = 12)	*p*-Value	90-Day Survivors (*n* = 27)	90-Day Decedents (*n* = 23)	*p*-Value
**Demographics**						
Age	67.58 ± 15.81	75.08 ± 9.56	*0.128*	65.00 ± 17.43	75.52 ± 8.91	*0.017*
Male	25 (65.8%)	8 (66.7%)	*1.000*	19 (70.4%)	14 (60.9%)	*0.480*
BMI	22.44 ± 4.65	24.84 ± 4.12	*0.115*	22.0 ± 5.09	24.19 ± 3.66	*0.094*
**Clinical characteristics**						
LOS to index culture, median (IQR)	46.50 (8.25, 113)	80.00 (31.75,149.75)	*0.345*	45.00 (5.00, 81.00)	61.00 (22.00, 142.00)	*0.101*
Charlson comorbidity index	5.63 ± 2.77	6.29 ± 4.14	*0.125*	5.59 ± 2.82	6.52 ± 3.55	*0.308*
Chemotherapy within previous 3 months	7 (18.4%)	5 (41.7%)	*0.129*	4 (14.8%)	8 (34.8%)	*0.099*
Steroid use within previous 3 months	17 (44.7%)	9 (75.0%)	*0.067*	13 (48.1%)	13 (56.5%)	*0.555*
Antibiotic use previous 3 months	36 (94.7%)	12 (100.0%)	*1.000*	26 (96.3%)	22 (95.7%)	*1.000*
Antifungal use previous 3 months	13 (34.2%)	6 (50.0%)	*0.496*	12 (44.4%)	7 (30.4%)	*0.309*
ICU care	17 (44.7%)	9 (75.0%)	*0.067*	11 (40.7%)	15 (65.2%)	*0.084*
ICU stay (IQR)	0 (0.00, 84.00)	47.50 (5.25, 110.75)	*0.101*	0 (0, 83.00)	36 (0, 87.00)	*0.101*
Ventilator care	19 (50.0%)	9 (75.0%)	*0.128*	13 (48.0%)	15 (65.2%)	*0.226*
HD/CVVH	15 (39.5%)	6 (50.0%)	*0.738*	10 (37.0%)	11 (47.8%)	*0.567*
Intravenous catheter	37 (97.4%)	11 (91.7%)	*1.000*	26 (96.3%)	22 (95.7%)	*1.000*
Neutropenia	1 (2.6%)	2 (16.7%)	*0.139*	1 (3.7%)	2 (8.7%)	*0.588*
Lymphopenia	23 (60.5%)	9 (81.8%)	*0.287*	16 (59.3%)	16 (72.7%)	*0.325*
**Severity of illness**						
Pitt score	3.5 ± 2.28	4.67 ± 2.77	*0.148*	3.11 ± 2.21	4.57 ± 2.48	*0.033*
SOFA score	6.29 ± 4.36	11.42 ± 5.50	*0.002*	5.4 ± 8.81	9.91 ± 5.46	*0.002*
**Admission source**						
Home	16 (42.1%)	4 (33.3%)	*0.740*	14 (51.9%)	6 (26.1%)	*0.064*
SNF	12 (31.6%)	3 (25.0%)	*1.000*	6 (22.2%)	9 (39.1%)	*0.193*
LTACH	10 (26.3%)	5 (41.7%)	*0.471*	7 (25.9%)	8 (34.8%)	*0.496*
**Colonization**						
*C. auris* colonization	14 (36.8%)	9 (75.0%)	*0.021*	11 (40.7%)	12 (52.5%)	*0.419*
Non *C. auris Candida* species colonization	10 (26.3%)	7 (58.3%)	*0.077*	8 (29.6%)	9 (39.1%)	*0.480*
*Candida albicans*	5 (13.2%)	4 (33.3%)	*0.191*	3 (11.1%)	6 (26.1%)	*0.270*
*Candida tropicalis*	7 (18.4%)	3 (25.0%)	*0.686*	5 (18.5%)	5 (21.7%)	*1.000*
*Candida glabrata*	1 (2.6%)	1 (8.3%)	*1.000*	1 (3.7%)	1 (4.3%)	*1.000*
*Candida parapsilosis*	1 (2.6%)	2 (16.7%)	*0.139*	0 (0.0%)	3 (13.0%)	*0.090*
*Candida orthopilosis*	0 (0.0%)	1 (8.3%)	*0.240*	0 (0.0%)	1 (4.3%)	*0.430*
CRO colonization	24 (64.9%)	7 (58.3%)	*0.521*	17 (63.0%)	14 (60.9%)	*1.000*
**Source of BSI**						
Catheter-related infection	28 (73.7%)	9 (75.0%)	*1.000*	19 (70.4%)	18 (78.3%)	*0.526*
Intraabdominal infection	3 (7.9%)	0 (0.0%)	*1.000*	3 (11.1%)	0 (0.0%)	*0.240*
Urinary tract infection	5 (13.2%)	0 (0.0%)	*0.319*	4 (14.5%)	1 (4.3%)	*0.357*
Pneumonia	2 (5.3%)	3 (25.0%)	*0.082*	2 (7.4%)	3 (13.0%)	*0.651*
**BSI status**						
Persistent BSI	13 (34.2%)	5 (41.7%)	*0.735*	8 (29.6%)	10 (43.5%)	*0.309*
Breakthrough BSI	9 (24.7%)	2 (16.7%)	*1.000*	6 (22.2%)	5 (21.7%)	*1.000*
Relapse	6 (15.8%)	1 (8.3%)	*1.000*	4 (14.8%)	3 (13.0%)	*1.000*
BSI resolution within 30 days	27 (71.1%)	3 (25.0%)	*0.007*	24 (88.9%)	11 (47.8%)	*0.002*
**Removal of CVC**						
Removed < 48 h	24 (63.2%)	5 (41.7%)	*0.314*	17 (63.0%)	12 (52.2%)	*0.567*
**Antifungal use**						
Proper antifungal in <48 h	22 (57.9%)	9 (75.0%)	*0.332*	18 (66.7%)	13 (56.5%)	*0.461*
**Prior surgery**						
Prior surgery in previous 90 days	7 (18.4%)	3 (25.0%)	*0.449*	5 (18.5%)	5 (21.7%)	*0.526*

Abbreviation: BMI, body mass index; BSI, bloodstream infection; CRO, carbapenem-resistant organisms; CVC, central venous catheter; HD/CVVH, hemodialysis/continuous veno-venous hemofiltration; ICU, intensive care unit; IQR, interquartile range; LTACH, long-term acute care hospital; LOS, length of stay; SOFA, sequential organ failure assessment; SNF, skilled nursing facility. Data are presented as mean ± standard deviation (SD) for continuous variables and *n* (%) for categorical variables. Italic *p* denotes the *p*-value.

**Table 2:** Factors associated with 30-day mortality and 90-day mortality: multivariable analysis.

	30-Day Mortality			90-Day Mortality		
	Unadjusted OR (95% CI)	Adjusted OR (95% CI)	*p *Value	Unadjusted OR (95% CI)	Adjusted OR (95% CI)	*p * Value
Age	1.05 (0.99–1.12)	1.09 (1.01–1.19)	*0.045*	1.06 (1.01–1.12)	1.08 (1.01–1.17)	*0.027*
SOFA score	1.19 (1.03–1.35)	1.12 (1.01–1.42)	*0.040*	1.19 (1.04–1.36)	1.17 (0.99–1.39)	*0.073*
Pitt bacteremia score	NA	NA	NA	1.27 (0.99–1.64)	0.96 (0.65–1.44)	*0.870*
BSI resolution within 30 days	0.19 (0.05–0.74)	0.11 (0.02–0.69)	*0.019*	NA	NA	NA
BSI resolution within 90 days	NA	NA	NA	0.13 (0.03–0.55)	0.17 (0.03–0.99)	*0.049*
Charlson comorbidity index	1.15 (0.94–1.40)	1.05 (0.81–1.35)	*0.714*	1.09 (0.91–1.31)	0.98 (0.98–1.39)	*0.832*
*Candida auris* colonization	3.70 (0.97–14.29)	1.85 (0.28–12.17)	*0.523*	NA	NA	NA

Abbreviation: BSI, bloodstream infection; SOFA, sequential organ failure assessment. Variables not included in the final multivariable model are shown as NA. Italic *p* denotes the *p*-value.

**Table 3:** Sensitivity analysis of predictors of 30-day and 90-day mortality.

	**30-Day Mortality**			**90-Day Mortality**		
	**Unadjusted OR (95% CI)**	**Adjusted OR (95% CI)**	***p*** **Value**	**Unadjusted OR (95% CI)**	**Adjusted OR (95% CI)**	***p*** **Value**
Age	1.05 (1.00–1.13)	1.09 (1.01–1.19)	*0.039*	1.06 (1.01–1.13)	1.09 (1.02–1.18)	*0.021*
SOFA score	1.18 (1.03–1.37)	1.13 (0.97–1.33)	*0.126*	1.19 (1.05–1.38)	1.12 (0.96–1.34)	*0.159*
BSI resolution within 30 days	0.19 (0.04–0.70)	0.15 (0.03–0.76)	*0.028*	NA	NA	NA
BSI resolution within 90 days	NA	NA	NA	0.13 (0.03–0.50)	0.16 (0.02–0.89)	*0.046*

Abbreviation: BSI, bloodstream infection; SOFA, sequential organ failure assessment. Variables not included in the final multivariable model are shown as NA. Italic *p* denotes the *p*-value.

## Data Availability

The data presented in this study are available from the corresponding author on reasonable request.

## Abbreviations

The following abbreviations are used in this manuscript:

AST	antifungal susceptibility testing
BMI	body mass index
BSI	bloodstream infection
*C. auris*	*Candida auris*
CCI	Charlson comorbidity index
CRO	carbapenem-resistant organism
CVC	central venous catheter
HD/CVVH	hemodialysis/continuous veno-venous hemofiltration
ICU	intensive care unit
IRB	institutional review board
LOS	length of stay
LTACH	long-term acute care hospital
MALDI-TOF MS	matrix-assisted laser desorption/ionization time-of-flight mass spectrometry
SNF	skilled nursing facility
SOFA	Sequential Organ Failure Assessment
